# Vitamin D-mediated tsRNA-07804 triggers mitochondrial dysfunction and suppresses non-small cell lung cancer progression by targeting CRKL

**DOI:** 10.1007/s00432-023-05586-1

**Published:** 2024-01-30

**Authors:** Yonggang Liang, Xiaoqiang Zhang, Jinhua Peng, Jing Liu, He Chen, Shanxian Guo

**Affiliations:** 1 Department of Thoracic Surgery, The Second Affiliated Hospital of Nanchang University, Nanchang, 330006 China; 2 Department of Pathology, The Second Affiliated Hospital of Nanchang University, Nanchang, 330006 China; 3 Key Laboratory of Molecular Medicine, The Second Affiliated Hospital of Nanchang University, Nanchang, 330006 China; 4 Thoracic Oncology Department, Jiangxi Cancer Hospital, Jiangxi Clinical Research Center for Cancer, 519 Beijing East Road, Nanchang, 330029 China; 5 JXHC Key Laboratory of Tumor Microenvironment and Immunoregulation, Jiangxi Cancer Hospital, Nanchang, China

**Keywords:** Vitamin D, Non-small cell lung cancer, Progression, Mitochondrial dysfunction, tsRNA-07804, CRKL

## Abstract

**Objective:**

tRNA-derived small RNAs (tsRNAs) are novel non-coding RNAs with various functions in multiple cancers. Nevertheless, whether vitamin D executes its function in mitochondrial dysfunction and non-small cell lung cancer (NSCLC) progression through tsRNAs remains obscure.

**Methods:**

Differentially expressed tsRNAs between control and vitamin D-treated H1299 cells were acquired by small RNA sequencing. Cell and animal experiments were implemented to elucidate the impacts of vitamin D and tsRNA on mitochondrial dysfunction and NSCLC progression. Dual-luciferase reporter assay, quantitative real-time PCR, western blot and recovery experiments were applied to determine the mechanism of tsRNA in NSCLC.

**Results:**

We discovered that vitamin D receptor resulted in decreased mitochondrial-related functions and vitamin D caused mitochondrial dysfunction of NSCLC cells. tsRNA-07804 was remarkably upregulated in vitamin D-treated H1299 cells. Functional experiments indicated that vitamin D led to mitochondrial dysfunction, repressed the proliferation, migration, invasion, and promoted apoptosis of H1299 cells via regulating tsRNA-07804. Mechanistically, tsRNA-07804 induced mitochondrial dysfunction and inhibited the malignancy of H1299 cells by suppressing CRKL expression. In vivo experiments showed that vitamin D inhibited the tumor growth in NSCLC by increasing tsRNA-07804 expression. Moreover, clinical sample analysis unveiled that tsRNA-07804 had a negative correlation with CRKL.

**Conclusions:**

In conclusion, our study proved that vitamin D induced mitochondrial dysfunction and suppressed the progression of NSCLC through the tsRNA-07804/CRKL axis. Overall, these results unveiled that tsRNA-07804 might act as a potential therapeutic target for NSCLC.

**Supplementary Information:**

The online version contains supplementary material available at 10.1007/s00432-023-05586-1.

## Introduction

Lung cancer is the second most familiar category of cancers globally and is deemed to be a dominant reason of cancer-associated deaths (Kielbowski et al. 2023). Non-small cell lung cancer (NSCLC) is the most familiar subtype that occupies 85–90% of all lung cancer types (Xu et al. 2023). NSCLC is a heterogeneous cancer characterized by a wide range of carcinogenic driver changes. Currently, treatment options for NSCLC principally include surgery, radiotherapy, chemotherapy, traditional Chinese medicine, immunotherapy, and antibody–drug conjugates (Chen et al. 2022). Although there have been great progresses in the treatment of NSCLC, the recurrence and metastasis rates reach up to 30–40%, and the 5-year overall survival rate is below 15% (Wang and Li 2018). In consequence, it is indispensable to further probe into the mechanism of the development of NSCLC and identify new therapeutic targets.

Vitamin D is generally deemed to be a key nutrient for calcium intake, especially for developing children and the elderly (Kubodera 2009). Vitamin D is indispensable for sustaining healthy mineral and bone metabolism. Vitamin D also play a vital part in arterial blood pressure, cardiovascular complications, immune responses, diabetes, and cancers (Urena-Torres and Souberbielle 2014). Vitamin D has been discovered to exert anti-tumor effects and be a predictor of prognosis in cancers (Santos and Hussain 2019; Urashima et al. 2019). Sufficient vitamin D levels were related with a decreased risk of sporadic colorectal cancer (Ma et al. 2023). Low vitamin D levels had correlation with poorer prognosis in primary melanomas (Timerman et al. 2017). Vitamin D curbed tumor growth and facilitated CD8+ T cell infiltration in breast cancer (Karkeni et al. 2019). Vitamin D was also reported to restrain tumor growth, migration, and proliferation by inhibiting HRC expression in lung cancer (Liu et al. 2021a). Mechanically, vitamin D binds to vitamin D receptor (VDR), forms dimers with retinol X receptor, and binds to vitamin D reaction elements in target gene promoters to form complexes, recruit co-regulators, and further modulate transcription of downstream genes (Bouillon et al. 2008; Fleet et al. 2012; Haussler et al. 2013; Bikle 2014; Chen et al. 2016). However, the specific mechanism of vitamin D in NSCLC remains vague.

tRNA-derived small RNAs (tsRNAs) represent a novel class of small non-coding RNAs that are vital for protein translation (Wang et al. 2023). tsRNAs mainly contain tRNA halves (tiRNAs) and tRNA-derived fragments (tRFs) (Li et al. 2018). A good deal of research has revealed the involvement of tsRNAs in tumorigenesis and pathogenesis of multiple cancers (Wang et al. 2022b). For instance, tRF-21-RKP4P9L0 was discovered to have a negative correlation with the prognosis of lung adenocarcinoma and tRF-21-RK9P4P9L0 silencing depressed biological malignant behavior of A549 and H1299 cell lines (Wang et al. 2022a). A research unveiled that upregulation of AS-tDR-007333 facilitated NSCLC cell proliferation and migration (Yang et al. 2022a). Another study found that tsRNA-5001a was connected with prognosis of lung adenocarcinoma patients and boosted the proliferation ability of lung adenocarcinoma cells (Hu et al. 2021). However, whether vitamin D regulates tsRNA expression to participate in NSCLC progression remains obscure.

In our study, we aimed to figure out the role and regulatory mechanism of vitamin D in mitochondrial dysfunction and NSCLC progression. We explored tsRNA expression profiles between control and vitamin D-treated H1299 cell using small RNA sequencing. Cell functional experiments and animal experiments was utilized to investigate the impact and mechanism of vitamin D-mediated tsRNA-07804 in mitochondrial dysfunction and NSCLC progression. Our study will offer novel molecular mechanisms and therapeutic method for the progression of NSCLC.

## Material and methods

### Clinical samples

A total of 16 pairs of tumor tissues and adjacent tissues were acquired from NSCLC patients who received surgery at the second affiliated hospital of Nanchang university from June 2021 to February 2022. Table S1 displayed clinical characteristics of all patients. All the collected samples were reserved at − 80 °C for the future analyses. This study was given permission by the Medical Ethics Review Committee of the Second Affiliated Hospital of Nanchang University. All NSCLC patients offered their informed consent.

### Bioinformatic analysis

The LinkedOmics database (https://www.linkedomics.org/admin.php) was used to analyze VDR expression-related genes in lung squamous carcinoma (LUSC) and lung adenocarcinoma (LUAD) (Vasaikar et al. 2018). The function of these genes was analyzed using Gene Set Enrichment Analysis (GSEA). Correlation analyses of VDR and CRKL expression was performed using the GEPIA database. The TNMplot database was used to analyze the expression of CRKL in LUSC tissues. The Kaplan–Meier algorithm was applied to analyze the disease prognostic markers, including overall survival (OS), first progression (FP), and post progression survival (PPS).

### H1299 cell culture and treatment

The human NSCLC cell line H1299 was purchased from Chinese Academy of Sciences Cell Bank (Beijing, China). These cells were authenticated by short tandem repeat method. The H1299 cells were cultured in RPMI-1640 medium (CORNING, 10–040-CVR, USA), which contained 10% fetal bovine serum (GIBCO, USA) and 1% penicillin/streptomycin (Shanghai, China) in a 5% CO_2_ incubator (3111, Thermo) at 37 °C. H1299 cells were treated with 1 × 10^–6^ mol/L of vitamin D (D1530, Sigma) for 96 h.

### Glucose uptake detection

The glucose concentrations in H1299 cells were detected using glucose uptake fluorometric assay kit (MAK084, Sigma-aldrich) according to the manufacturer’s instructions.

### ATP detection

The ATP content in H1299 cells was examined using the ATP assay kit (TW30551, Shanghai Tongwei Biotechnology Co., LTD) according to the manual provided by the producer. The OD values were assayed at a wavelength of 700 nm.

### Lactic acid detection

The lactic acid content in H1299 cells was examined using the lactic acid assay kit (D799099-0100, Sangon Biotech) according to the manual provided by the producer. The OD values were measured at a wavelength of 570 nm.

### Detection of mitochondrial membrane potential

The mitochondrial membrane potential in H1299 cells was assessed by the JC-1 detection kit (C2006, Beyotime, China). The cells were added with JC-1 dyeing solution and incubated at 37 °C for 20 min. Subsequently, cells were washed twice with JC-1 staining buffer. Finally, the green and red fluorescence intensity was observed by fluorescence microscopy (OLYMPUS, CKX53).

### Measurement of mitochondrial activity

H1299 cells were grown in 12-well plates overnight. Subsequently, the medium was discarded, and cells were incubated in Mito-Tracker Green solution for 30 min at 37 °C. At the end of incubation, Mito-Tracker Green solution was removed and fresh cell medium was appended. Stained cells were observed under a fluorescence microscopy (OLYMPUS, CKX53).

### Measurement of mitochondrial ROS

MitoSox Red probe was utilized to examine the content of mitochondrial ROS in H1299 cells. H1299 cells were treated with 5 μM MitoSox Red at 37 °C for 30 min. The fluorescence intensity was assayed under the microscope (OLYMPUS, CKX53).

### Small RNA sequencing

Total RNA was separated from the control and vitamin D-treated H1299 cells with TRIzol (Invitrogen, USA). The quality and concentration of RNA were examined by a NanoDrop ONE (Thermo Fisher Scientific, USA). RNA integrity was evaluated by 2% agarose gel electrophoresis. The libraries were acquired by employing the Multiplex Small RNA Library Prep Kit (USA). In brief, ligand primers were appended to both ends of the RNA fragments and cDNA construction was conducted using PCR. Afterwards, PCR products were separated by 8% SDS-PAGE. Small RNA libraries were quantified with Qubit 3.0 (Invitrogen, USA), and the insertion fragment size of the library was assayed with Agilent 2200 Bioanalyzer (USA). Small RNA sequencing was executed utilizing the Illumina HiSeq X Ten platform (USA). In the process of tsRNA analysis, rRNA was aligned to sequences that were not aligned to miRBase (12–23 bp, 34–43 bp) and piRNAcluster (24–33 bp). After filtering out sequences that could be aligned to rRNA, the remaining sequences were aligned to the GtRNA database, and then aligned to the tRFdb and tRFMINTbase database to obtain tsRNA. The R package DEseq2 was applied to acquire differentially expressed tsRNAs (DE-tsRNAs) on the base of |log2 (fold change)|> 1 and FDR < 0.05.

### Target prediction and annotation

The miRanda and RNAHybrid were employed to predict the targeted genes of  DE-tsRNAs. In the end, the combination of the two databases was regarded as the results of target genes prediction. The biological function target genes were unveiled by GO enrichment analysis. Pivotal signaling pathways were determined by KEGG enrichment analysis. Cytoscape v3.6.0 was applied for the construction of the network of tsRNA-target gene-signaling pathways. The criterion for remarkable GO terms and KEGG pathways is *P* value less than 0.05.

### Cell transfection

H1299 cells were inoculated into 6-well plates with 30 × 10^4^ cells/well. For the upregulation and downregulation of tsRNA-07804, tsRNA-07804 mimics and inhibitor were transfected into H1299 cells by employing Lipofectamine™ 2000 (Invitrogen) following the manual offered by the manufacturer.

### Quantitative real-time PCR (qRT-PCR)

Total RNAs were separated from H1299 cells with Trizol reagent (Invitrogen) following the manufacturer's manual. The concentration and purity of total RNAs were evaluated by a TGem spectrophotometer (TIANGEN). RNA integrity was determined by agarose gel electrophoresis (EPS300, Shanghai Tianneng Technology Co., LTD, China). Next, RevertAid First Strand cDNA Synthesis Kit (Thermo#K1622) was implemented to synthesize cDNAs. qRT-PCR was implemented by employing FastStart Universal SYBR Green Master (Roche). The expression of each gene was assessed with the 2^−ΔΔCt^ method. Table S2 displayed primer sequences.

### Cell counting kit-8 (CCK-8) assay

Cell proliferation of H1299 cells was assayed by CCK-8. H1299 cells were treated with vitamin D and then transfected with tsRNA-07804 inhibitor/mimics. Treated cells were inoculated into 6-well plates and cultured in 5% CO2 incubator at 37 °C overnight. Afterwards, CCK-8 reagent was appended. After 1 h, the absorbance was tested with a microplate reader (Infinite M1000, TECAN) at 450 nm.

### Transwell

H1299 cells were treated with vitamin D and then transfected with tsRNA-07804 inhibitor/mimics. For cell migration experiment, H1299 cells (1 × 10^6^ cells/mL) were appended to the upper chamber. RPMI 1640 medium containing 10% serum was appended to the lower chamber. After 24 h of incubation, the H1299 cells were fixed with paraformaldehyde for 30 min, followed by crystal violet (Beyotime) staining. For invasion experiment, the upper chamber was precoated with BioCoat™ Matrigel (354,480, BioCoat). Other experimental steps referred to the migration experiment. The migrated and invasive cells were observed with a microscope (XSP-37XB, Shanghai, China).

### Flow cytometry

After treating H1299 cells with vitamin D and tsRNA-07804 inhibitor/mimics for 48 h, the cells were rinsed with PBS, and resuspended in 1 × Binding Buffer. Cells were subsequently treated with Annexin V-FITC in the dark at room temperature for 15 min. After washing with 1 × Binding Buffer, cellular staining was conducted with propidium lodide. A flow cytometer (FACSVerseTM) was adopted to detect the apoptosis rate of H1299 cells.

### Western blot

H1299 cells were lysed with RIPA buffer (Thermo). Proteins were segregated by SDS-PAGE and then transferred to polyvinylidene fluoride membranes. The membranes were blocked by TBST solution with 5% skim milk and subsequently incubated with primary antibodies against CRKL (1:1000, ab32018, Abcam), p16 (1:5000, 10883-1-AP, Proteintech), p21 (1:2000, 10355–1-AP, 10355–1-AP), and GAPDH (1:2000, 60004-1-Lg, Proteintech). In the end, membranes were incubated with a specific secondary antibody (1:1000, ab205719, Abcam) and visualized by ECL Blotting Detection Reagents (Thermo). Protein quantification was carried out using ImageJ software.

### Dual-luciferase reporter assay

Dual-luciferase reporter assay was adopted to detect the regulation of tsRNA-07804 on CRKL 3'UTR. CRKL wild type, CRKL mutant type containing tsRNA-07804 binding site were constructed and cloned into psiCHECK-2 plasmid. Subsequently, 293 T cells reached 70–80% confluence and subsequently co-transfected with either CRKL wild type or CRKL mutant luciferase reporter vector and either mimic tsRNA-07804 or negative control. After 48 h, luciferase activity was assayed.

### Nude mice xenograft experiments

A total of 15 female BALB/c nude mice (4–6 weeks) were from SPF (Beijing) Biotechnology Co., Ltd. (China). The mice were allocated to 3 groups: blank group, vitamin D+NC group, and vitamin D+tsRNA-07804 knockdown group (5 mice per group). All mice were given free access to food and water under specific pathogen-free conditions to acclimate for 7 days before the experiment. Mice in vitamin D+NC group and vitamin D+tsRNA-07804 knockdown group received 10000 IU/kg vitamin D3. After 7 weeks, 5 × 10^6^ H1299 cells stable transfected with LV3(H1/GFP&Puro)-tsRNA-07804 inhibitor sponge to knockdown tsRNA-07804 or control lentiviral were inoculated into mice subcutaneously. Tumor size was examined every 3 days. Mice were sacrificed, and tumors were collected after 4 weeks. The animal experiments were conducted following the Medical Ethics Review Committee of Nanchang University.

### Immunohistochemistry

Xenograft tumor tissues collected were fixed in 4% paraformaldehyde, followed by embedding. The embedded tissues were sliced to obtain 4 μm thick sections. After dewaxing, and antigen retrieval, the slides were put into 3% hydrogen peroxide and then blocked with 5% BSA. The slides were incubated with the primary antibody anti-CRKL (1:100, ab32018, Abcam, UK) overnight, whereafter incubated with a secondary antibody (G1215-200 T, Servicebio) for 50 min at room temperature. The sections were counterstained with hematoxylin and dehydrated. Finally, images were obtained under a microscope.

### Statistical analysis

GraphPad Prism Version 9.0 software (USA) was adopted for data analysis. All data were displayed as mean ± standard deviation. Student’s *t* test and one-way analysis of variance was utilized to compare the significance between two groups or among multiple groups, respectively. Values of *P* < 0.05 were deem to be statistically significant.

## Results

### Vitamin D induces mitochondrial dysfunction of NSCLC cells

**Fig. 1:**
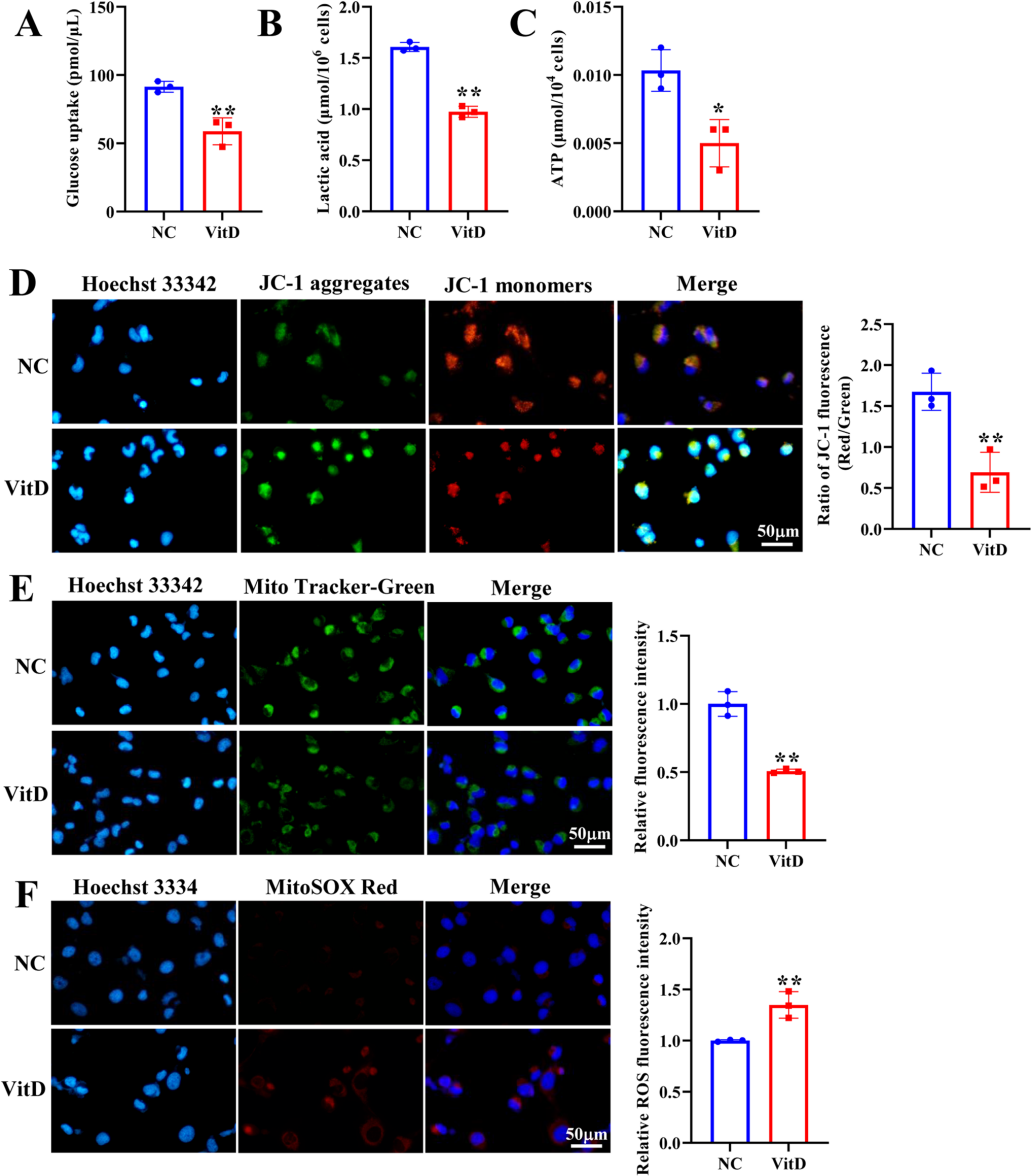
Vitamin D induces mitochondrial dysfunction of NSCLC cells. **A** Glucose uptake, **B** lactate production, and **C** ATP production were detected in H1299 cells treated with vitamin D. **D** The effect of vitamin D on mitochondrial membrane potential was determined by JC-1 fluorescent probe. **E** The effect of vitamin D on mitochondrial content was determined by Mito-Tracker Green staining. **F** The effect of vitamin D on mitochondrial ROS was determined by MitoSOX staining. Scale bar = 50 μm. *NC* normal control group, *VitD* Vitamin D treatment group. *P < 0.05, **P < 0.01

Vitamin D mainly regulates the transcription of downstream genes by binding to VDR and forming dimer with retinol X receptor. To explore the mechanism of vitamin D regulation in NSCLC, we used the LinkedOmics database (https://www.linkedomics.org/admin.php) to analyze VDR expression-related genes in LUSC and LUAD, and further analyzed the function of these genes. Results showed that VDR was positively or negatively correlated with multiple genes in LUSC (Fig. S1A, B) and LUAD (Fig. S2A, B). It was also found that VDR resulted in decreased mitochondrial-related functions in LUSC (Fig. S1C–E) and LUAD (Fig. S2C, D). To further clarify the function of vitamin D in mitochondrial dysfunction of NSCLC cells, H1299 cells were treated with vitamin D. Vitamin D-treated H1299 cells displayed decreased glucose uptake, lactic acid production, and ATP production (Fig. 1A–C). JC-1 staining revealed that vitamin D prominently decreased in red fluorescence intensity, whereas increased green fluorescence intensity in H1299 cells (Fig. 1D). Mito-Tracker Green staining demonstrated that vitamin D markedly reduced mitochondrial content in H1299 cells (Fig. 1E). MitoSOX staining results uncovered that vitamin D markedly increased the levels of mitochondrial ROS (Fig. 1F). Overall, vitamin D led to mitochondrial dysfunction of NSCLC cells.

### Vitamin D modulates tsRNA expression profiles in NSCLC cells

**Fig. 2:**
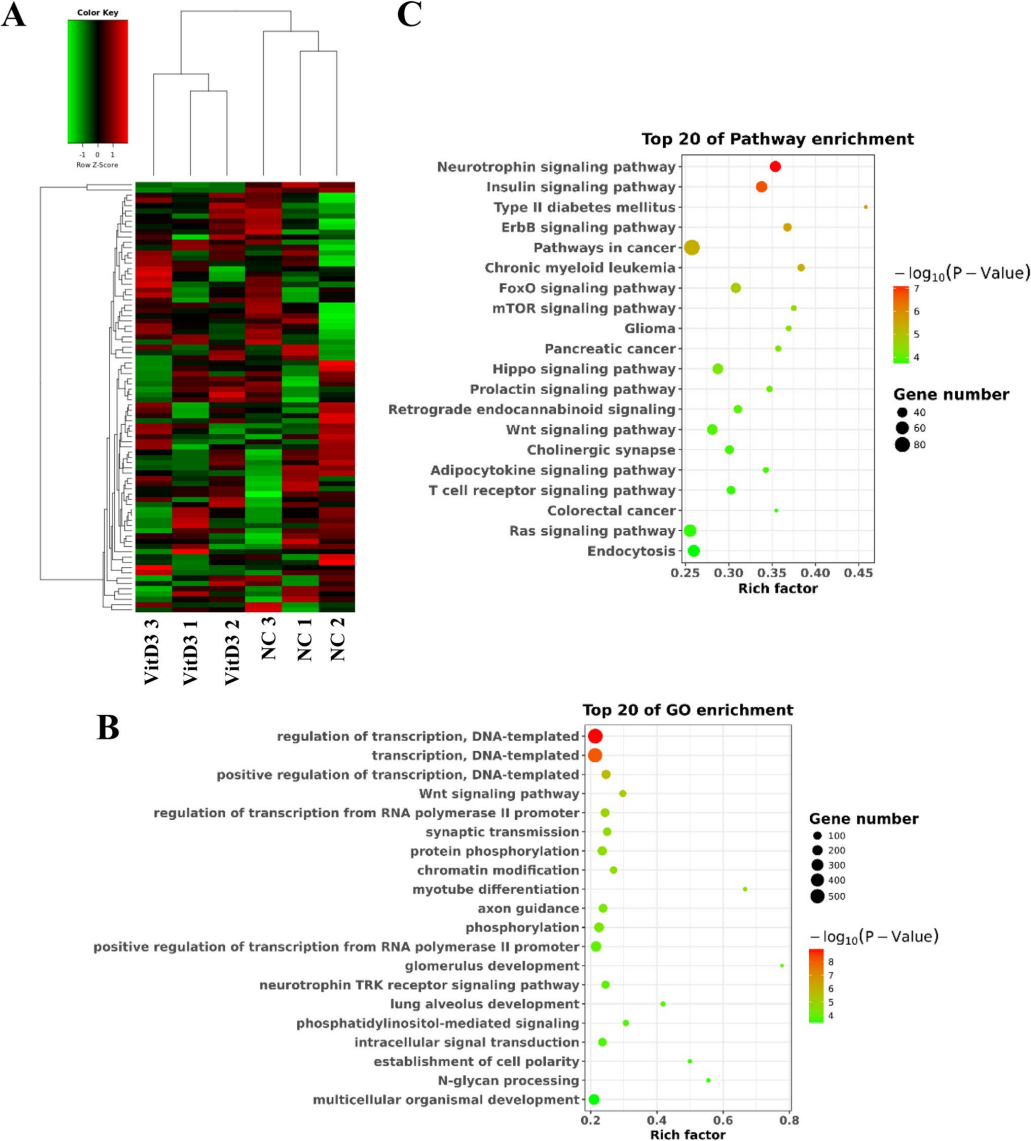
Analysis of tsRNA expression and related function in vitamin D-treated NSCLC cells. **A** Heatmap displayed differentially expressed tsRNAs in H1299 cells of vitamin D treatment and control groups. GO (**B**) and KEGG enrichment analysis (**C**) of vitamin D-regulated differentially expressed tsRNAs

We then investigated the molecular mechanism by which vitamin D regulated mitochondrial function in NSCLC. Since tsRNA is a novel functional RNA molecule and has been reported to be associated with mitochondrial function (Meseguer 2021), we carried out small RNA sequencing in vitamin D-treated and control H1299 cells and determined the key tsRNA involved in vitamin D regulation of mitochondrial function. It was found that 82 tsRNAs were differentially expressed in vitamin D-treated H1299 cells compared with control cells. Among them, 80 tsRNAs were markedly upregulated and 2 tsRNAs were remarkably downregulated in the vitamin D treatment group (Fig. 2A). To elucidate the potential function of DE-tsRNAs, we performed functional enrichment and pathway analysis of DE-tsRNAs target genes. GO functional analysis indicated that target genes of DE-tsRNAs were principally involved in transcriptional regulation, synaptic conduction, protein phosphorylation and other biological functions (Fig. 2B). KEGG enrichment analysis showed that target genes of DE-tsRNAs chiefly participated in Insulin, ErbB, cancer, and FoxO signaling pathways (Fig. 2C).

### Vitamin D causes mitochondrial dysfunction and restrains malignant phenotypes of NSCLC cells by regulating tsRNA-07804

**Fig. 3:**
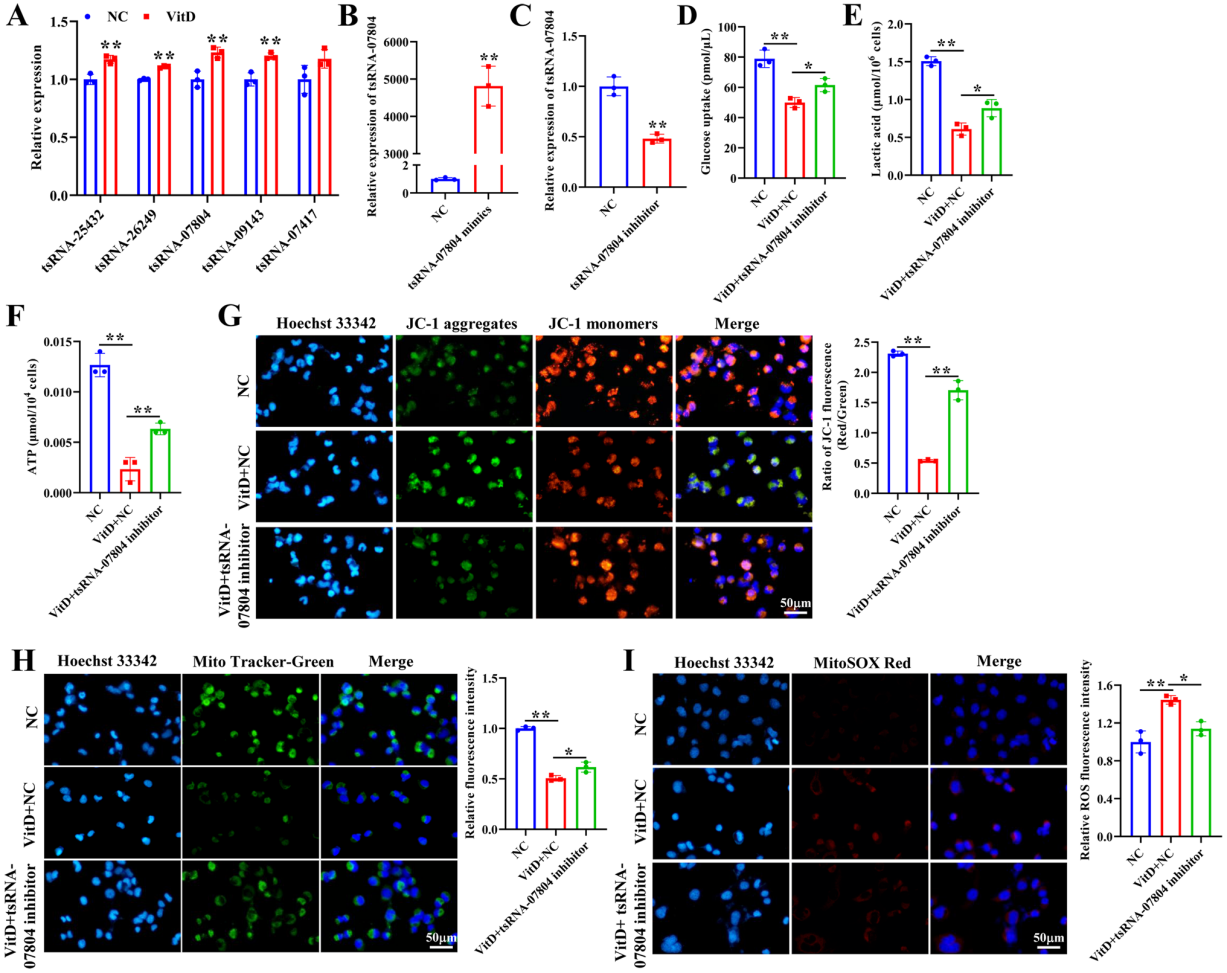
Vitamin D upregulated tsRNA-07804 to cause mitochondrial dysfunction of NSCLC cells. **A** qRT-PCR analysis of expression of five candidate tsRNAs in vitamin D-treated H1299 cells. **B**, **C** qRT-PCR analysis of overexpression and interference efficiency of tsRNA-07804 in H1299 cells. **D** Glucose uptake, **E** lactate production, and **F** ATP production were detected in in vitamin D-treated H1299 cells transfected with tsRNA-07804 inhibitor. **G** The effect of knockdown of tsRNA-07804 on mitochondrial membrane potential in vitamin D-treated H1299 cells was determined by JC-1 fluorescent probe. **H** The effect of knockdown of tsRNA-07804 on mitochondrial content in vitamin D-treated H1299 cells was determined by Mito-Tracker Green staining. **I** The effect of knockdown of tsRNA-07804 on mitochondrial ROS in vitamin D-treated H1299 cells was determined by MitoSOX staining. Scale bar = 50 μm.*P < 0.05, **P < 0.01

**Fig. 4:**
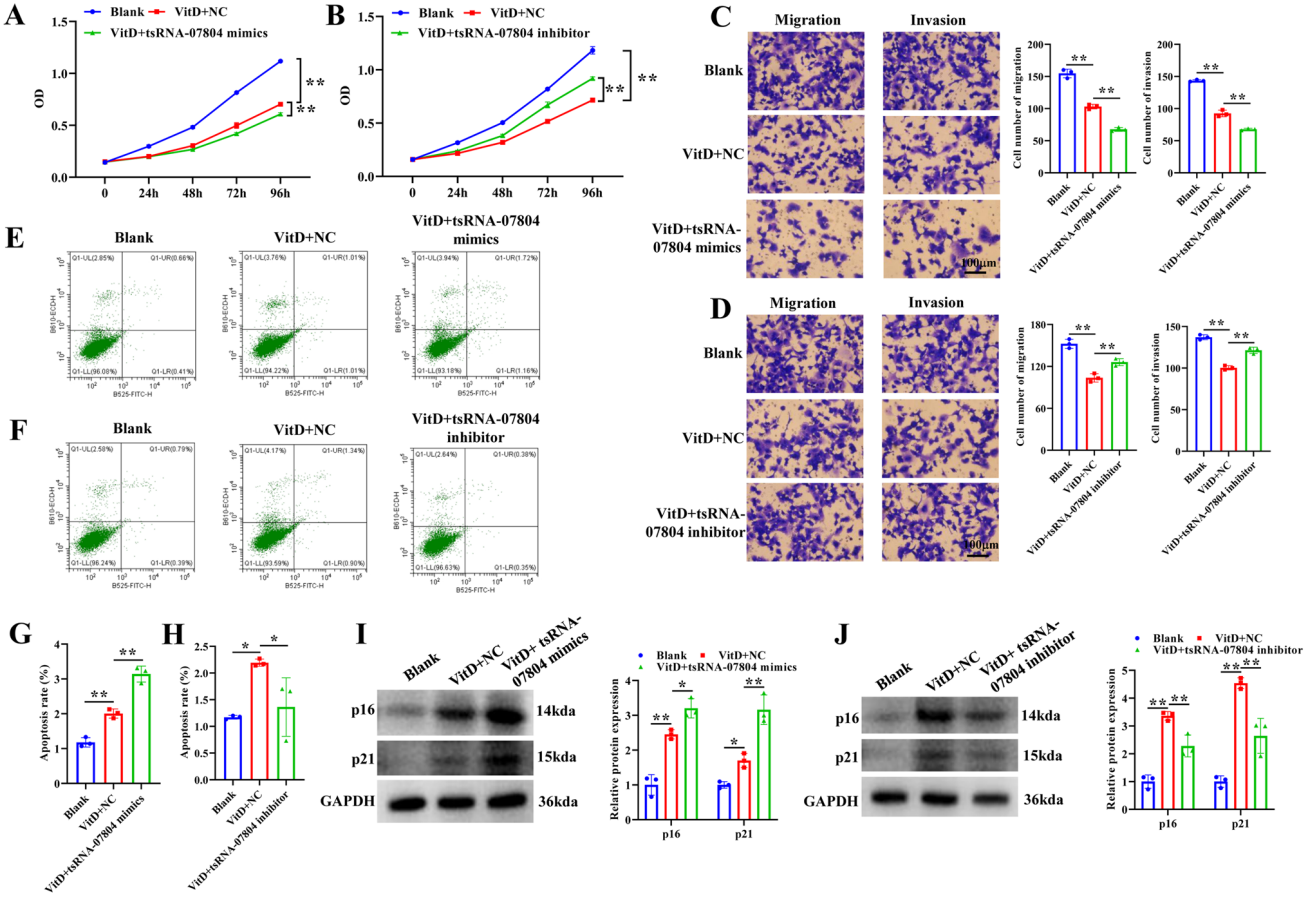
Vitamin D upregulated tsRNA-07804 to inhibit malignant activities of NSCLC cells. **A** The impact of vitamin D and tsRNA-07804 overexpression on the cell viability of H1299 cells was assayed by CCK-8. **B** The effects of vitamin D and tsRNA-07804 interference on the proliferation of H1299 cells were assayed by CCK-8. **C** The effects of vitamin D and overexpression of tsRNA-07804 on migration and invasion of H1299 cells were measured by transwell assay. Scale bar = 100 μm. **D** The effects of vitamin D and tsRNA-07804 interference on H1299 cell migration and invasion were assayed via the transwell assay. Scale bar = 50 μm. **E** The effect of vitamin D and overexpression of tsRNA-07804 on apoptosis of H1299 cells was determined via flow cytometry. **F** The impact of vitamin D and tsRNA-07804 interference on H1299 cell apoptosis was assayed by flow cytometry. **G**, **H** Quantitative analysis of apoptosis regulated by tsRNA-07804 overexpression or knockdown. **I**, **J** The protein expression of senescence markers in tsRNA-07804 knockdown H1299 cells treated with vitamin D was detected by western blot. *P < 0.05, **P < 0.01

To screen tsRNAs that participated in mitochondrial dysfunction and the progression of NSCLC, we screened five DE-tsRNAs (tsRNA-25432, tsRNA-26249, tsRNA-07804, tsRNA-09143, tsRNA-07417) according to the signaling pathways that were closely related to tumor progression (Fig. S3). qRT-PCR results showed that tsRNA-07804 was dramatically increased in the vitamin D group, with the largest fold change (Fig. 3A). Therefore, tsRNA-07804 was selected for subsequent study. To investigate the impact of tsRNA-07804 on mitochondrial dysfunction of NSCLC cells, we transfected tsRNA-07804 mimics and inhibitors into H1299 cells. As displayed in Fig. 3B and C, the expression of tsRNA-07804 was remarkably increased and decreased after tsRNA-07804 overexpression or knockdown, respectively. Vitamin D markedly suppressed glucose uptake, lactic acid production, and ATP production of H1299 cells, whereas tsRNA-07804 knockdown reversed this trend (Fig. 3D–F). JC-1 staining revealed that tsRNA-07804 inhibition obviously abolished the inhibitory effect of vitamin D on red fluorescence intensity in H1299 cells (Fig. 3G). Mito-Tracker Green staining demonstrated that vitamin D markedly reduced mitochondrial content in H1299 cells, which was abolished by silence of tsRNA-07804 (Fig. 3H). MitoSOX staining results revealed that tsRNA-07804 inhibition obviously inhibited the elevated levels of mitochondrial ROS by vitamin D (Fig. 3I). In addition, we investigated the impact of tsRNA-07804 on vitamin D-treated NSCLC cell behaviors. CCK-8 assay showed that vitamin D prominently reduced the proliferation of H1299 cells, and overexpression of tsRNA-07804 further confined the proliferation of vitamin D-treated H1299 cells (Fig. 4A). However, tsRNA-07804 knockdown obviously counteracted the suppressive effect of vitamin D on proliferation of H1299 cells (Fig. 4B). Transwell results displayed that vitamin D obviously diminished the migration and invasion of H1299 cells, and on this basis, further inhibition of the migration and invasion of H1299 cells was achieved through overexpression of tsRNA-07804 (Fig. 4C). However, tsRNA-07804 interference abolished the repressive effect of vitamin D on migration and invasion of H1299 cells (Fig. 4D). Flow cytometry displayed that vitamin D significantly facilitated H1299 cell apoptosis, and on this basis, overexpression of tsRNA-07804 further promoted H1299 cell apoptosis (Fig. 4E, G). On the contrary, silence of tsRNA-07804 showed the opposite results (Fig. 4F, H). Western blot revealed that vitamin D remarkably elevated the protein expression of senescence markers, p16 and p21 in H1299 cells, and on this basis, tsRNA-07804 overexpression further promoted their expression (Fig. 4I). Conversely, tsRNA-07804 knockdown showed the opposite results (Fig. 4J). Taken together, vitamin D caused mitochondrial dysfunction and limited the malignant phenotypes of NSCLC cells by modulating tsRNA-07804.

### tsRNA-07804 negatively regulates CRKL

**Fig. 5:**
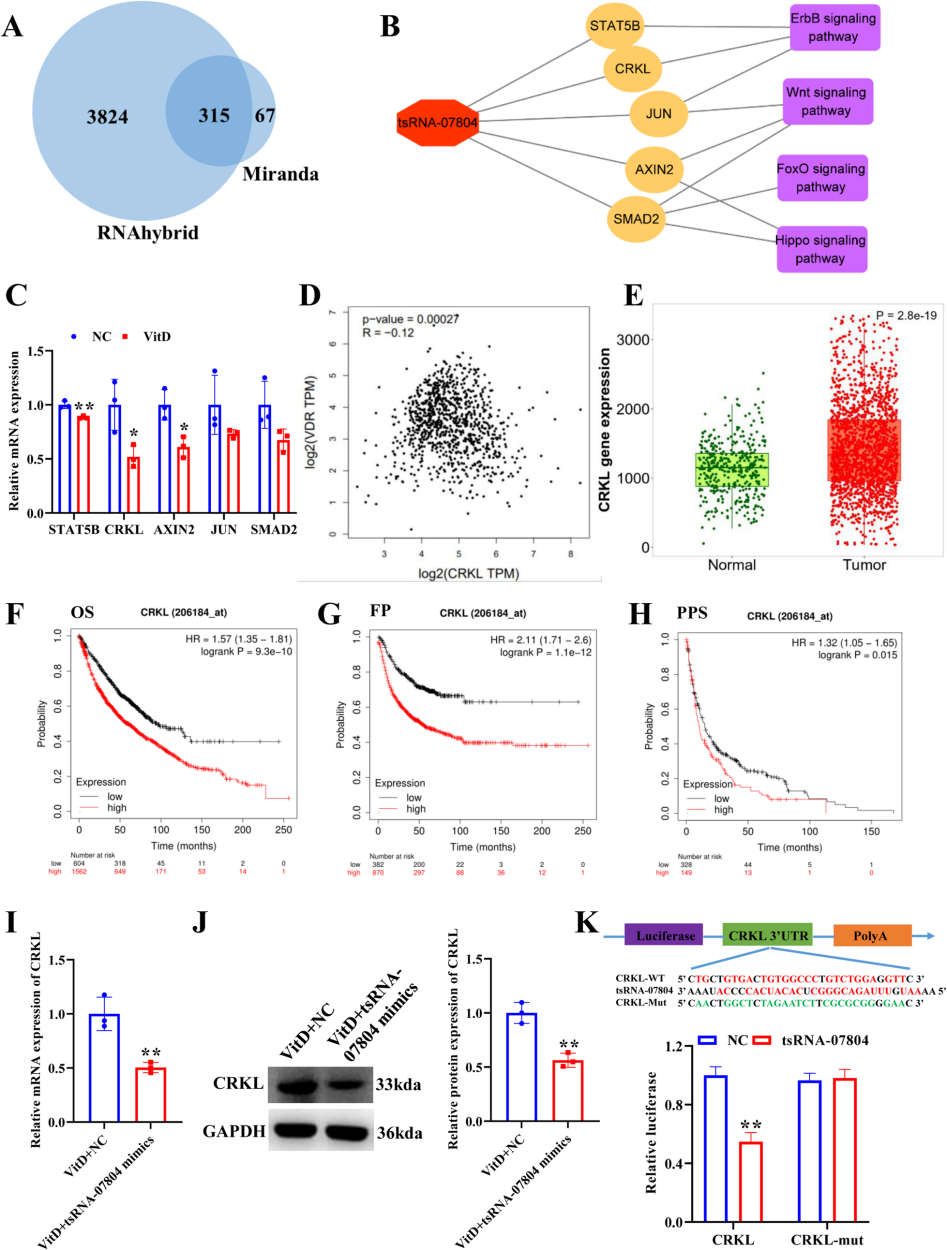
tsRNA-07804 negatively regulates CRKL. **A** Venn diagram showing target genes regulated by tsRNA-07804. **B** The network of tsRNA-07804-target genes-signaling pathways. **C** qRT-PCR analysis of the relative expression of five candidate target genes of tsRNA-07804. **D** Correlation analyses of VDR expression and CRKL expression in the GEPIA database. **E** The expression of CRKL in LUSC tissues using the TNMplot database. **F**–**H** Correlation analyses of CRKL expression and NSCLC patients’ overall survival (OS), first progression (FP), and post progression survival (PPS). The expression of CRKL in H1299 cells after overexpression of tsRNA-07804 was assayed by qRT-PCR (**I**) and western blot (**J**). **K** Dual-luciferase activity detection. *P < 0.05, **P < 0.01

To excavate the molecular mechanism of tsRNA-07804 in modulating the biological behavior of NSCLC cells, we utilized miRanda and RNAhybrid to predict the downstream target genes regulated by tsRNA-07804. The results of tsRNA-07804 target gene prediction analysis are shown in Fig. 5A. RNAhybrid analysis showed 4139 target genes, miranda analysis showed 382 target genes, and the two analysis shared 315 target genes. Five genes (STAT5B, CRKL, AXIN2, JUN, SMAD2) were selected as alternative target genes of tsRNA-07804 (Fig. 5B) because these target genes are involved in signaling pathways (ErbB, Wnt, FoxO, and Hippo signaling pathways) related to mitochondrial function (Farhan et al. 2017; Sigismund et al. 2018; Delgado-Deida et al. 2020; Liu et al. 2021b). Subsequently, we treated H1299 cells with vitamin D and measured the expression of alternative target genes (STAT5B, CRKL, AXIN2, JUN, and SMAD2) via qRT-PCR. The results uncovered that CRKL was prominently decreased in vitamin D-treated H1299 cells with the largest fold change (Fig. 5C). Therefore, we selected CRKL as the target gene of tsRNA-07804. Correlation analysis showed that VDR was negatively correlated with CRKL (Fig. 5D). TNMplot data analysis results showed that CRKL was remarkably upregulated in tumor tissues of LUSC patients compared with non-tumor tissues (Fig. 5E). Kaplan–Meier curves showed that the NSCLC patients with low CRKL expression had remarkably better OS, FP and PPS (Fig. 5F–H). Next, we further explored the relation between tsRNA-07804 and CRKL. Overexpression of tsRNA-07804 remarkably inhibited the mRNA and protein expression of CRKL in vitamin D-treated H1299 cells (Fig. 5I, J). We verified the interaction between tsRNA-07804 and CRKL by utilizing the dual-luciferase reporting assay. As displayed in Fig. 5K, tsRNA-07804 had a remarkable repressive effect on the expression of CRKL-3’UTR-WT luciferase reporter, whereas did not affect CRKL-3’-UTR-Mut luciferase reporter expression. Overall, tsRNA-07804 downregulated the expression of CRKL.

### tsRNA-07804 triggers mitochondrial dysfunction and inhibits malignant activities of NSCLC cells by restraining CRKL expression

**Fig. 6:**
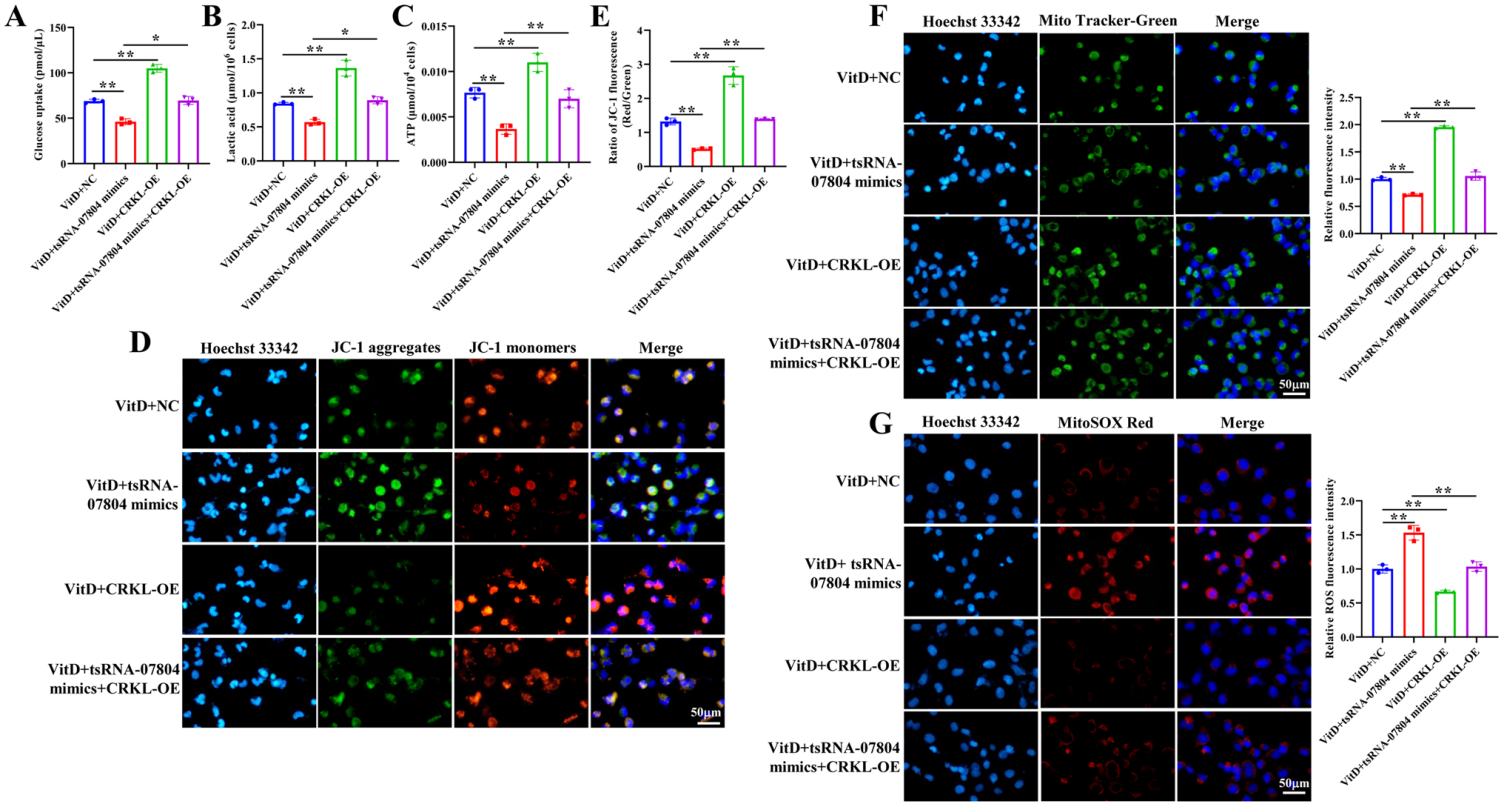
tsRNA-07804 results in mitochondrial dysfunction of NSCLC cells via downregulating CRKL expression. The effect of CRKL overexpression on **A** glucose uptake, **B** lactate production, and **C** ATP production in tsRNA-07804 overexpressed H1299 cells were assessed by corresponding kits. **D** The effect of CRKL overexpression on mitochondrial membrane potential in tsRNA-07804 overexpressed H1299 cells was determined by JC-1 fluorescent probe. **E** Quantitative analysis of JC-1 staining. **F** The effect of CRKL overexpression on mitochondrial content in tsRNA-07804 overexpressed H1299 cells was determined by Mito-Tracker Green staining. **G** The effect of CRKL overexpression on ROS in tsRNA-07804 overexpressed H1299 cells was determined by MitoSOX staining. Scale bar = 50 μm. CRKL-OE represents CRKL overexpression. *P < 0.05, **P < 0.01

**Fig. 7:**
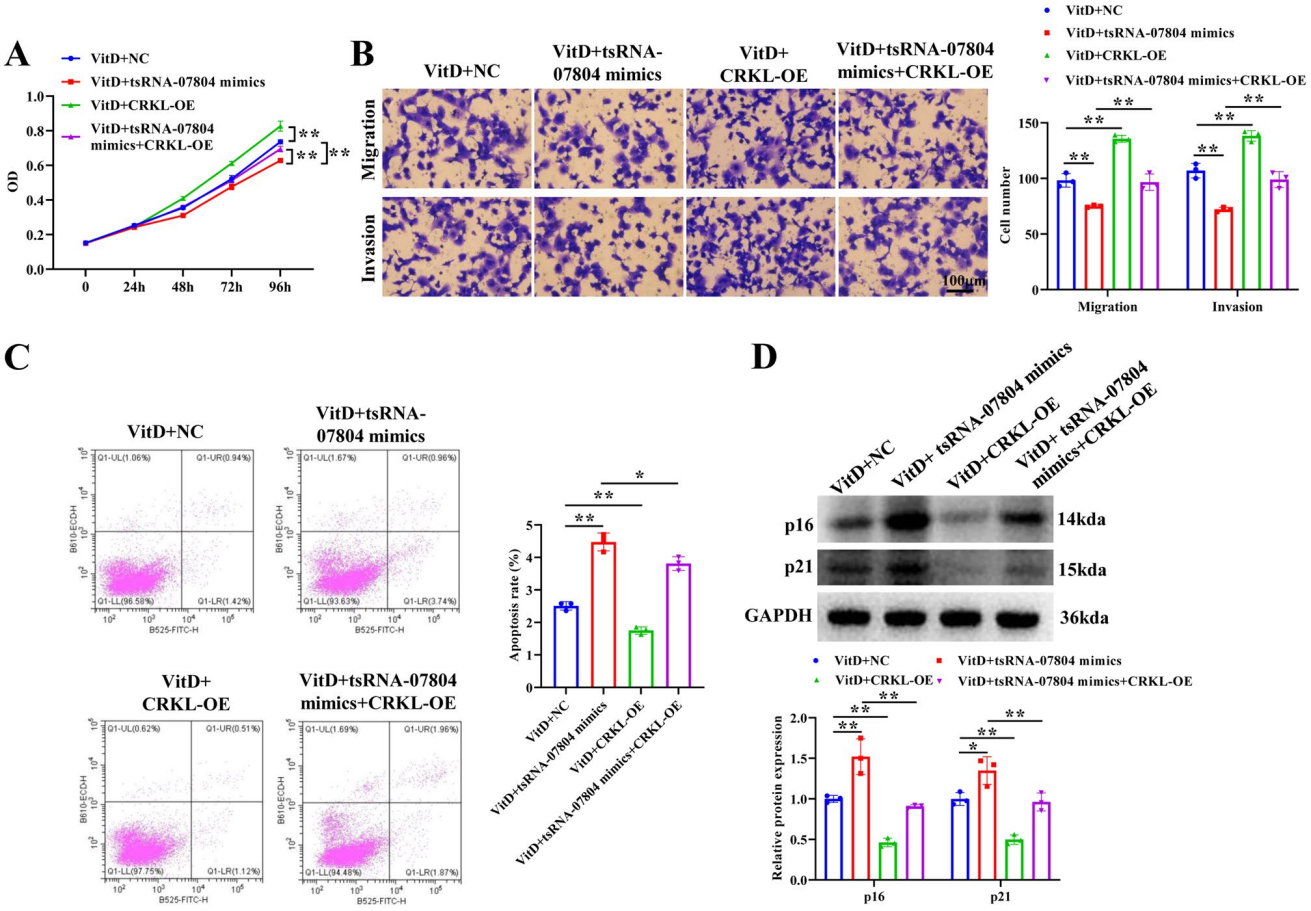
tsRNA-07804 suppresses malignant behaviors of NSCLC cells via downregulating CRKL expression. Impacts of CRKL overexpression on proliferation (**A**), migration and invasion (**B**), and apoptosis (**C**) in tsRNA-07804 overexpressed H1299 cells were assayed by CCK-8, transwell, and flow cytometry, respectively. Scale bar = 100 μm. **D** The protein expression of senescence markers in tsRNA-07804 overexpressed and CRKL overexpressed H1299 cells was detected by western blot. *P < 0.05, **P < 0.01

To investigate whether tsRNA-07804 affects the malignant behaviors of NSCLC cells through CRKL, H1299 cells were transfected with tsRNA-07804 mimics and the CRKL overexpression vector. The findings of qRT-PCR and western blot indicated that overexpression of tsRNA-07804 notably inhibited the mRNA and protein expression of CRKL in vitamin D-treated H1299 cells, while overexpression of CRKL reversed this trend (Fig. S4A, B). We then investigated whether tsRNA-07804 leads to mitochondrial dysfunction by regulating CRKL. We discovered that tsRNA-07804 overexpression decreased glucose uptake, lactic acid production, and ATP production in H1299 cells, while these effects were reversed by CRKL overexpression (Fig. 6A–C). JC-1 staining revealed that the red fluorescence intensity was noticeably decreased in tsRNA-07804-overexpressed H1299 cells and this ability was partly recovered when CRKL was overexpressed (Fig. 6D, E. Mito-Tracker Green staining demonstrated that tsRNA-07804 overexpression lessened mitochondrial content, which was recovered following CRKL overexpression (Fig. 6F). MitoSOX staining revealed that CRKL overexpression obviously repressed the elevated levels of mitochondrial ROS by tsRNA-07804 overexpression (Fig. 6G). Moreover, we also investigated whether tsRNA-07804 repressed malignant activities of NSCLC cells by regulating CRKL. As displayed in Fig. 7A and B, overexpression of tsRNA-07804 significantly inhibited, whereas CRKL overexpression remarkably augmented proliferation capacity, migration and invasion of H2199 cells. Of note, overexpression of CRKL could partially abolished the repressive effect of overexpression of tsRNA-07804 on proliferation, migration and invasion of H1299 cells. Flow cytometry unveiled that overexpression of tsRNA-07804 obviously augmented, while upregulation of CRKL strikingly declined the apoptosis of H2199 cells, and overexpression of CRKL could partially revoke the promotion of overexpression of tsRNA-07804 on the apoptosis of H1299 cells (Fig. 7C). Western blot revealed that CRKL overexpression remarkably suppressed tsRNA-07804 overexpression-caused increased protein expression of p16 and p21 in H1299 cells (Fig. 7D). Therefore, tsRNA-07804 induced mitochondrial dysfunction and repressed malignant activities of NSCLC cells by repressing CRKL expression in the presence of vitamin D.

### Vitamin D represses tumor growth via the tsRNA-07804/CRKL axis

**Fig. 8:**
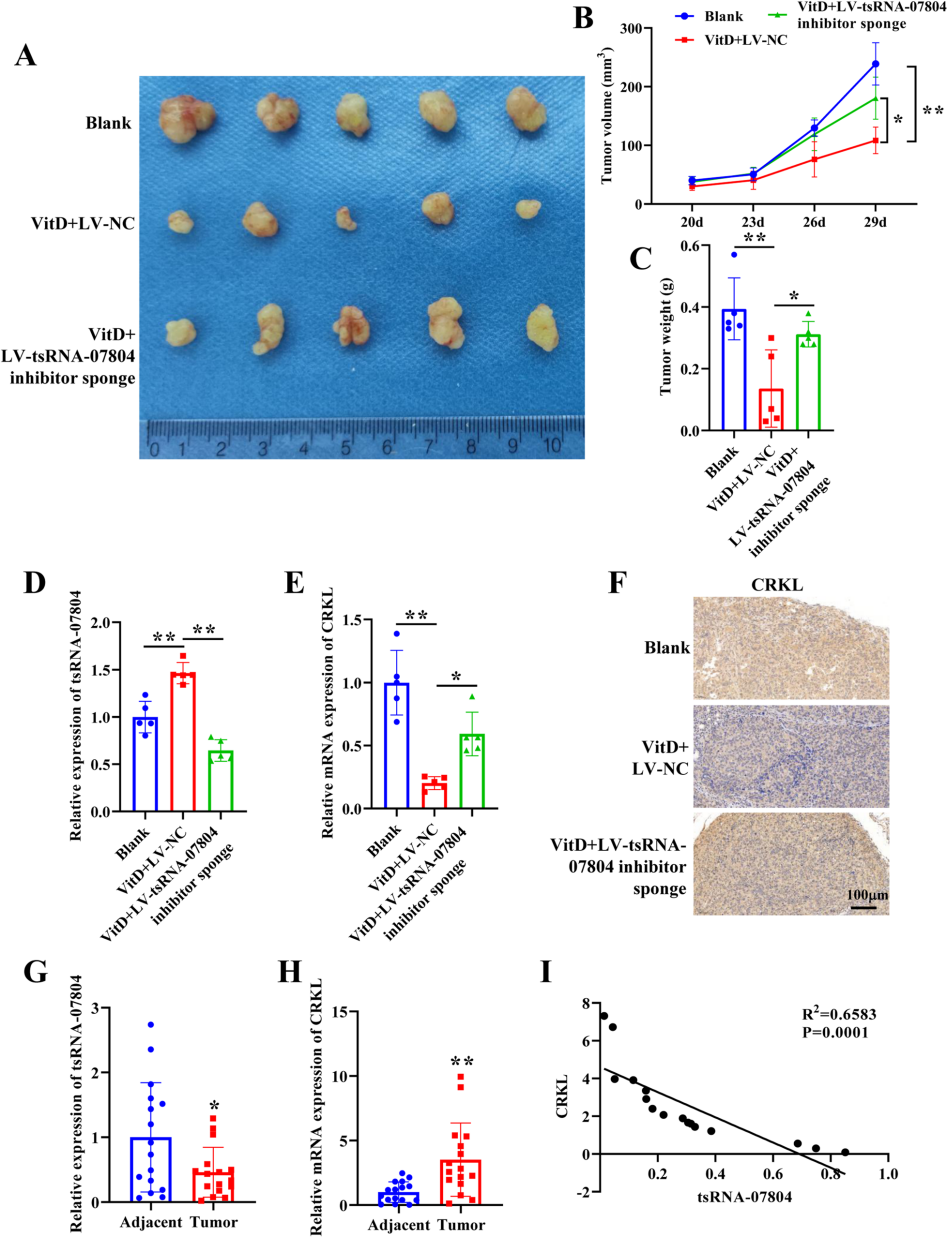
Vitamin D inhibits tumor growth via the tsRNA-07804/CRKL axis. **A** Images of tumors in different treatment groups. **B** Effects of vitamin D and tsRNA-07804 interference on tumor volume. **C** Effects of vitamin D and tsRNA-07804 interference on tumor weight. **D** qRT-PCR was applied to evaluate the effect of vitamin D and tsRNA-07804 interference on the expression of tsRNA-07804. **E** The impact of vitamin D and tsRNA-07804 interference on CRKL expression was assayed via qRT-PCR. **F** The effect of vitamin D and tsRNA-07804 interference on CRKL expression was assayed by immunohistochemistry. Scale bar = 100 μm. **G**, **H** tsRNA-07804 and CRKL expression in tumor tissues and normal adjacent tissues of NSCLC patients was tested through qRT-PCR (*n* = 16). **I** Correlation between tsRNA-07804 and CRKL. *P < 0.05, **P < 0.01

To figure out whether vitamin D plays a part in vivo through the regulation of tsRNA-07804, subcutaneous xenograft experiments were conducted. Compared with the control group, vitamin D remarkably inhibited tumor growth, while tsRNA-07804 knockdown partially restored tumor growth (Fig. 8A–C). qRT-PCR analysis uncovered that vitamin D significantly promoted the expression of tsRNA-07804 and curbed the expression of CRKL, while tsRNA-07804 knockdown partially reversed this trend (Fig. 8D, E). Immunohistochemistry confirmed the same results (Fig. 8F). Moreover, we evaluated the expression of tsRNA-07804 and CRKL in tumor tissues and adjacent tissues from patients with NSCLC (*n* = 16). As displayed in Fig. 8G, H, tsRNA-07804 was obviously downregulated, while CRKL was prominently upregulated in NSCLC tissues relative to the adjacent normal tissues. Moreover, tsRNA-07804 had negative connection with CRKL (Fig. 8I). Collectively, these results implied that vitamin D triggers mitochondrial dysfunction and inhibits the progression of NSCLC in vivo via the tsRNA-07804/CRKL axis (Fig. S5).

## Discussion

Vitamin D is a steroid hormone and vitamin D deficiency can resulted in risk of numerous cancers (Carlberg and Munoz 2022). Our objective was to reveal the role of vitamin D in NSCLC via regulating tsRNAs. In our study, we discovered that tsRNA-07804 was dramatically elevated in H1299 cells treated with vitamin D. Vitamin D induced mitochondrial dysfunction, and diminished the proliferation, migration, invasion, facilitated apoptosis of H1299 cells through upregulating tsRNA-07804 expression. Mechanistically, tsRNA-07804 induced mitochondrial dysfunction and inhibited the malignancy of H1299 cells by suppressing CRKL expression. In vivo experiments indicated that vitamin D inhibited the tumor growth in NSCLC by increasing tsRNA-07804 expression. Clinical sample analysis uncovered that tsRNA-07804 was conversely correlated with CRKL. These results indicate that tsRNA-07804 might serve as a prospective therapeutic target for NSCLC.

Accumulating research has shown that tsRNAs are aberrantly expressed lots of cancers and function in lots of cellular activities, including proliferation, apoptosis, invasion and metastasis, and are associated with prognosis of cancers (Pekarsky et al. 2023). Evidence has suggested that tsRNA-26576 expression was dramatically enhanced in breast cancer tissues, and tsRNA-26576 overexpressing enhanced growth of breast cancer cells (Zhou et al. 2019). 5ʹ-tiRNA-Gln overexpression curbed the development and progression of hepatocellular carcinoma (Wu et al. 2023). Our findings indicated that vitamin D diminished proliferation, migration and invasion of NSCLC cells through enhancing tsRNA-07804 expression. Additionally, mitochondrial dysfunction has been deemed to be a hallmark of cancer (Hsu et al. 2016). Yang et al. discovered that CLDN10 overexpression restrained clear cell renal cell carcinoma cell proliferation, migration and invasion and caused mitochondrial dysfunction (Yang et al. 2022b). Chan et al. demonstrated that genistein induce mitochondrial dysfunction and promoted apoptosis in NSCLC cells (Chan et al. 2022). In agreement with these findings, tsRNA-07804 triggered mitochondrial dysfunction of NSCLC cells. Collectively, our study demonstrated that tsRNA-0780 caused mitochondrial dysfunction and inhibited malignant phenotypes in NSCLC.

To elaborate the mechanism of tsRNA-07804 in NSCLC, we focused on the signaling pathways that target genes of tsRNA-07804 were involved in. We discovered that target genes of tsRNA-07804 principally participated in ErbB, FoxO, and Hippo signaling pathways. These pathways were closely associated with the development of cancer. ERBB was discovered to be responsible for increased cell proliferation and migration in glioblastoma multiforme (Gheidari et al. 2022). CTCF silencing diminished the progression of prostate cancer via altering the FoxO signalling pathway (Shan et al. 2019). The Hippo signaling pathway was also reported to modulate cancer cell proliferation, invasion, and migration (Mohajan et al. 2021). Evidence showed that interference of HMGA2 retarded migration, invasion and metastasis by repressing the Hippo-YAP signaling pathway in breast cancer (Xu et al. 2021). Taken together, tsRNA-07804 may exert a suppressive role in the development of NSCLC via ErbB, FoxO, and Hippo signaling pathways.

Evidence has shown that tsRNAs can inhibit the expression of target genes by directly binding to the 3’ UTRs of target genes. For example, tRFdb-3003a/b was found to restrain VAV2 expression via binding to the 3’UTR regions of VAV2 in gliomas (Ren et al. 2022). tiRNA-Gly-GCC-1 was discovered to diminish TLR4 expression via directly targeting 3ʹUTR of TLR4 in urothelial bladder carcinoma (Qin et al. 2022). The present study revealed that tsRNA-07804 targeted CRKL and overexpression of tsRNA-07804 suppressed CRKL expression. CRKL belongs to the CRK adapter protein family and participated in the development and progression of many cancers (Sriram and Birge 2010; Shi et al. 2015). A previous study has reported that overexpression and interference of CRKL strikingly facilitated and curbed migration and invasion abilities of hepatocellular carcinoma HepG2 cells (Guo et al. 2018). Evidence has suggested that CRKL silencing dramatically suppressed bladder cell proliferation and migration (Liu et al. 2019). In gastric cancer, CRKL was markedly correlated with clinicopathologic features, and inhibition of CRKL diminished gastric cancer cell proliferation (Wang et al. 2013). In lung cancer, overexpression of CRKL facilitated cell invasion via ERK-MMP9 pathway (Lin et al. 2015). CRKL overexpression was also discovered to promote cell proliferation, migration and invasion in lung adenocarcinoma (Liu et al. 2021c). CRKL was also reported to enhance the glucose metabolism of cancer cells by activating PI3K/Akt, thus promoting the occurrence of hepatocarcinoma (Guo et al. 2021). In accordance with the above findings, our study pointed out that CRKL overexpression inhibited mitochondrial dysfunction and promoted NSCLC malignancy.

In conclusion, our research sheds light on the tumor-suppressive effect of tsRNA-07804 in NSCLC. We unveiled that vitamin D induced mitochondrial dysfunction and restrained the progression of NSCLC via the tsRNA-07804/CRKL axis. tsRNA-07804 exerted inverse correlation with CRKL in the aspect of clinical sample analysis. Overall, targeting tsRNA-07804 may be a hopeful therapeutic approach for NSCLC.

### Supplementary Information

Below is the link to the electronic supplementary material.

Supplementary file1 Fig. S1 VDR is involved in mitochondrial function in LUSC. (A) VDR was positively correlated with multiple genes in LUSC. (B) VDR was negatively correlated with multiple genes in LUSC. VDR expression-related genes were enriched in (C) mitochondrial respiratory chain complex assembly, (D) mitochondrial gene expression, and (E) mitochondrial RNA metabolic process. VDR represents vitamin D receptor. LUSC represents lung squamous carcinoma. (TIF 11399 KB)

Supplementary file2 Fig. S2 VDR is involved in mitochondrial function in LUAD. (A) VDR was positively correlated with multiple genes in LUAD. (B) VDR was negatively correlated with multiple genes in LUAD. VDR expression-related genes were enriched in (C) mitochondrial gene expression and (D) mitochondrial RNA metabolic process. LUAD represents lung adenocarcinoma. (TIF 13065 KB)

Supplementary file3 Fig. S3 The network of candidate tsRNAs-target genes-signaling pathways. (TIF 7445 KB)

Supplementary file4 Fig. S4. Overexpression of CRKL recovers the expression of CRKL in H1299 cells transfected with tsRNA-07804 mimics. The effect of tsRNA-07804 overexpression and CRKL overexpression on CRKL expression were assayed by qRT-PCR (A) and western blot (B). *P<0.05, **P<0.01. (TIF 855 KB)

Supplementary file5 Fig. S5. Schematic depiction of the rle and regulatory mechanism of vitamin D-mediated tsRNA-07804 in NSCLC. (TIF 1261 KB)

Supplementary file6 (DOCX 14 KB)

Supplementary file7 (DOCX 14 KB)

## Data Availability

The datasets supporting the conclusions of this article are available from the corresponding author upon reasonable request.
