# Supplementary figures and images for: Vitamin D-mediated tsRNA-07804 triggers mitochondrial dysfunction and suppresses non-small cell lung cancer progression by targeting CRKL
Source: J Cancer Res Clin Oncol. 2024 Jan 30;150(2):51. doi: 10.1007/s00432-023-05586-1 (PMC10827823; doi:10.1007/s00432-023-05586-1)

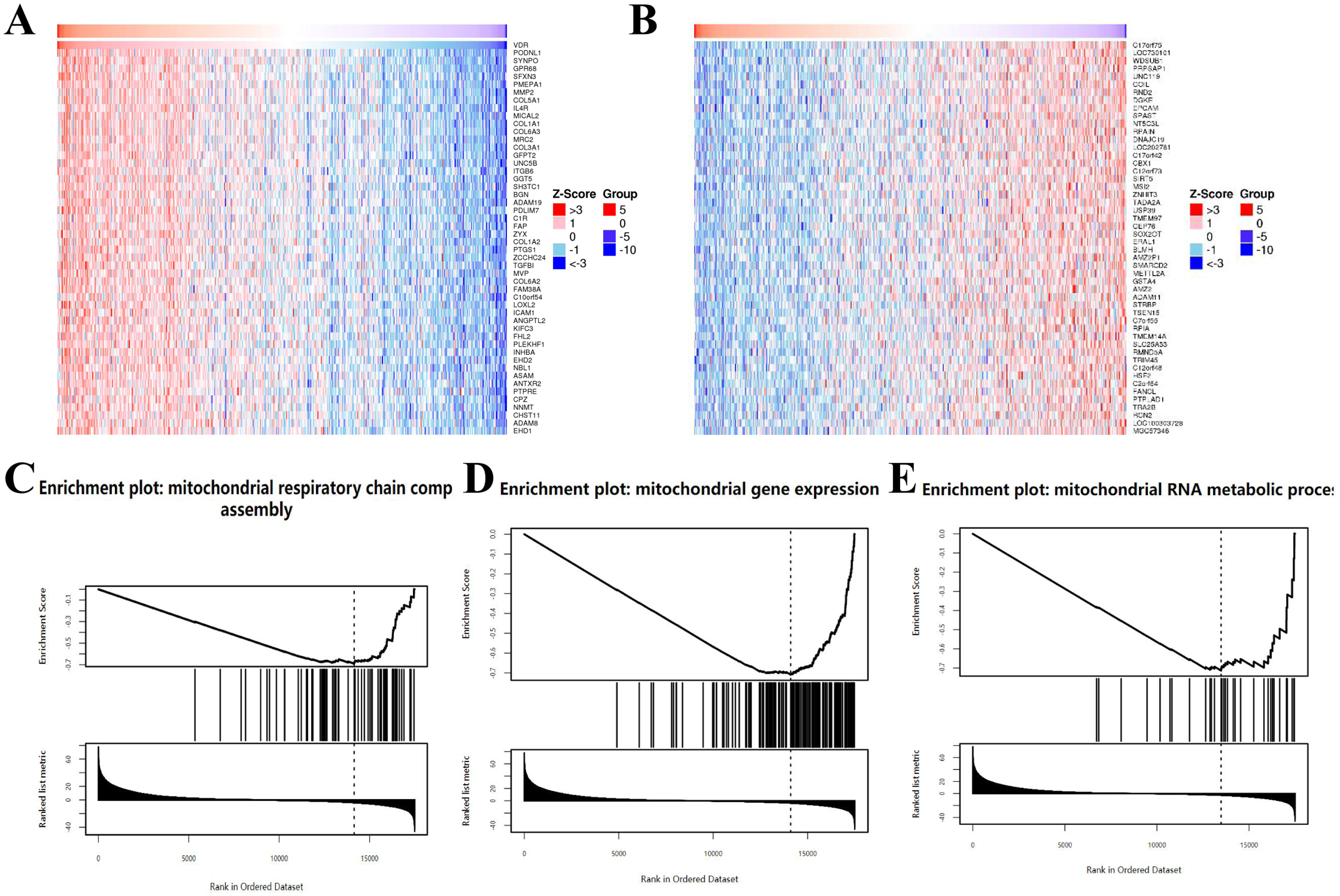

Supplement: Supplementary file 1 — Supplementary file1 Fig. S1 VDR is involved in mitochondrial function in LUSC. (A) VDR was positively correlated with multiple genes in LUSC. (B) VDR was negatively correlated with multiple genes in LUSC. VDR expression-related genes were enriched in (C) mitochondrial respiratory chain complex assembly, (D) mitochondrial gene expression, and (E) mitochondrial RNA metabolic process. VDR represents vitamin D receptor. LUSC represents lung squamous carcinoma. (TIF 11399 KB) [file 432_2023_5586_MOESM1_ESM.tif]

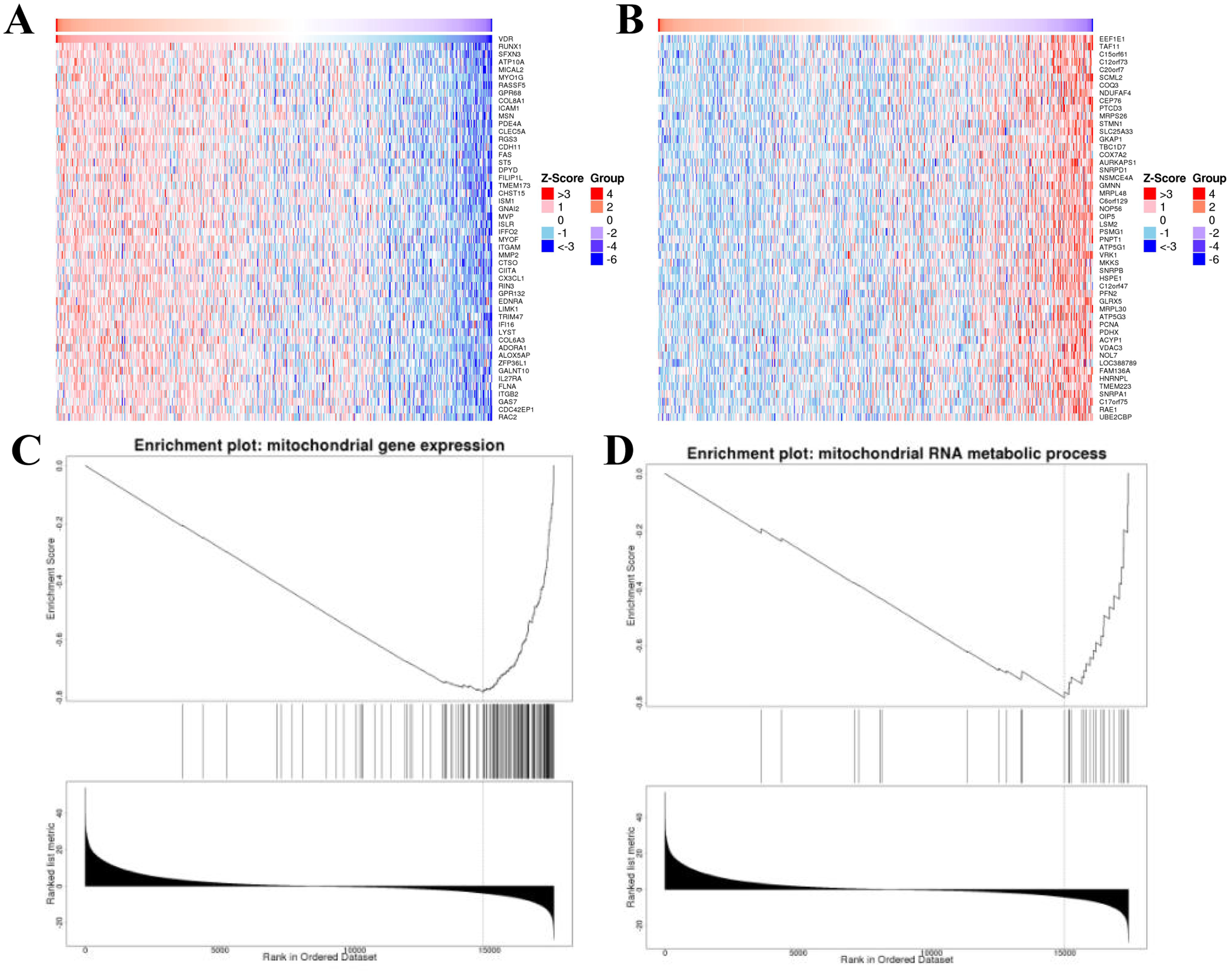

Supplement: Supplementary file 2 — Supplementary file2 Fig. S2 VDR is involved in mitochondrial function in LUAD. (A) VDR was positively correlated with multiple genes in LUAD. (B) VDR was negatively correlated with multiple genes in LUAD. VDR expression-related genes were enriched in (C) mitochondrial gene expression and (D) mitochondrial RNA metabolic process. LUAD represents lung adenocarcinoma. (TIF 13065 KB) [file 432_2023_5586_MOESM2_ESM.tif]

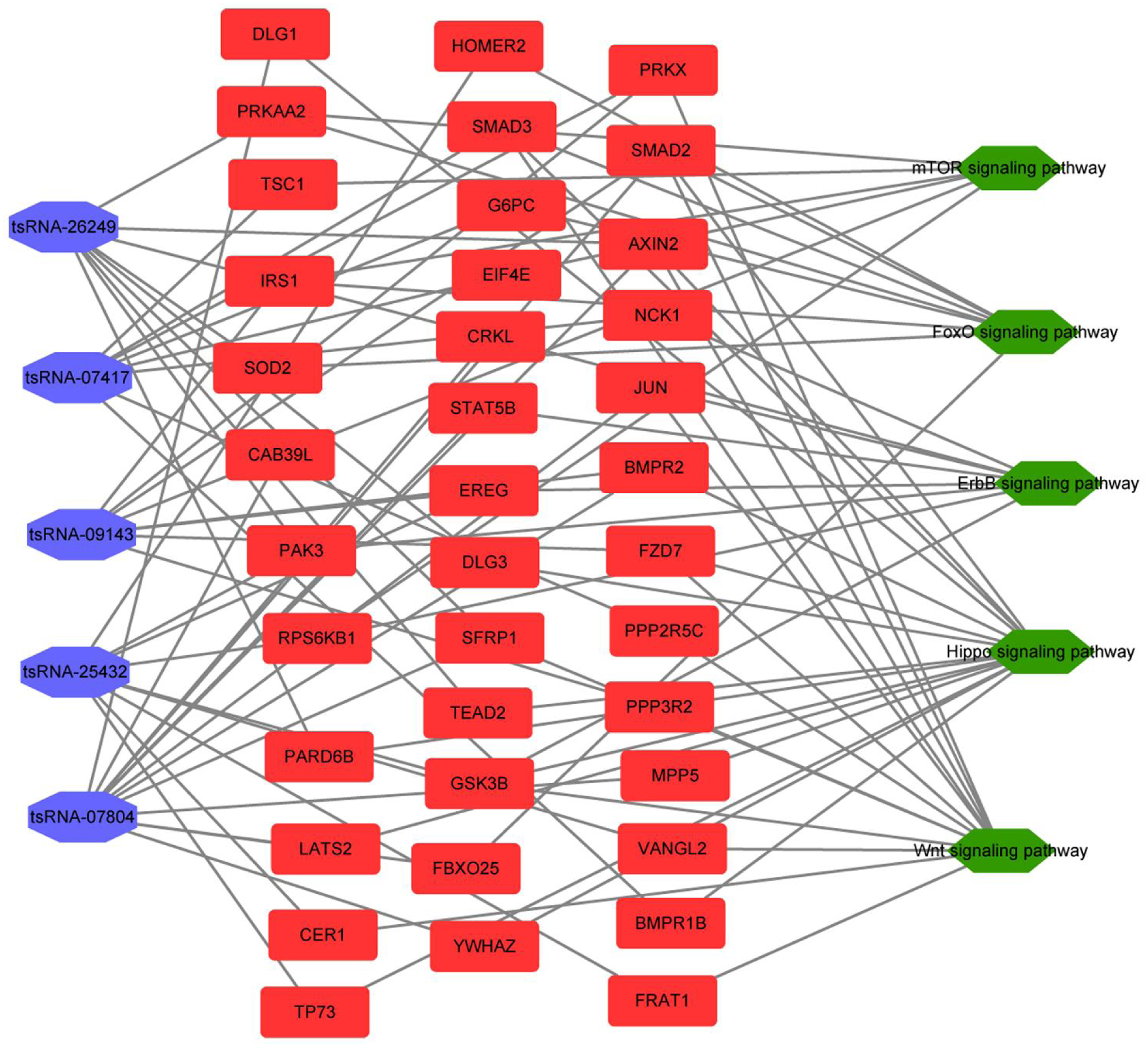

Supplement: Supplementary file 3 — Supplementary file3 Fig. S3 The network of candidate tsRNAs-target genes-signaling pathways. (TIF 7445 KB) [file 432_2023_5586_MOESM3_ESM.tif]

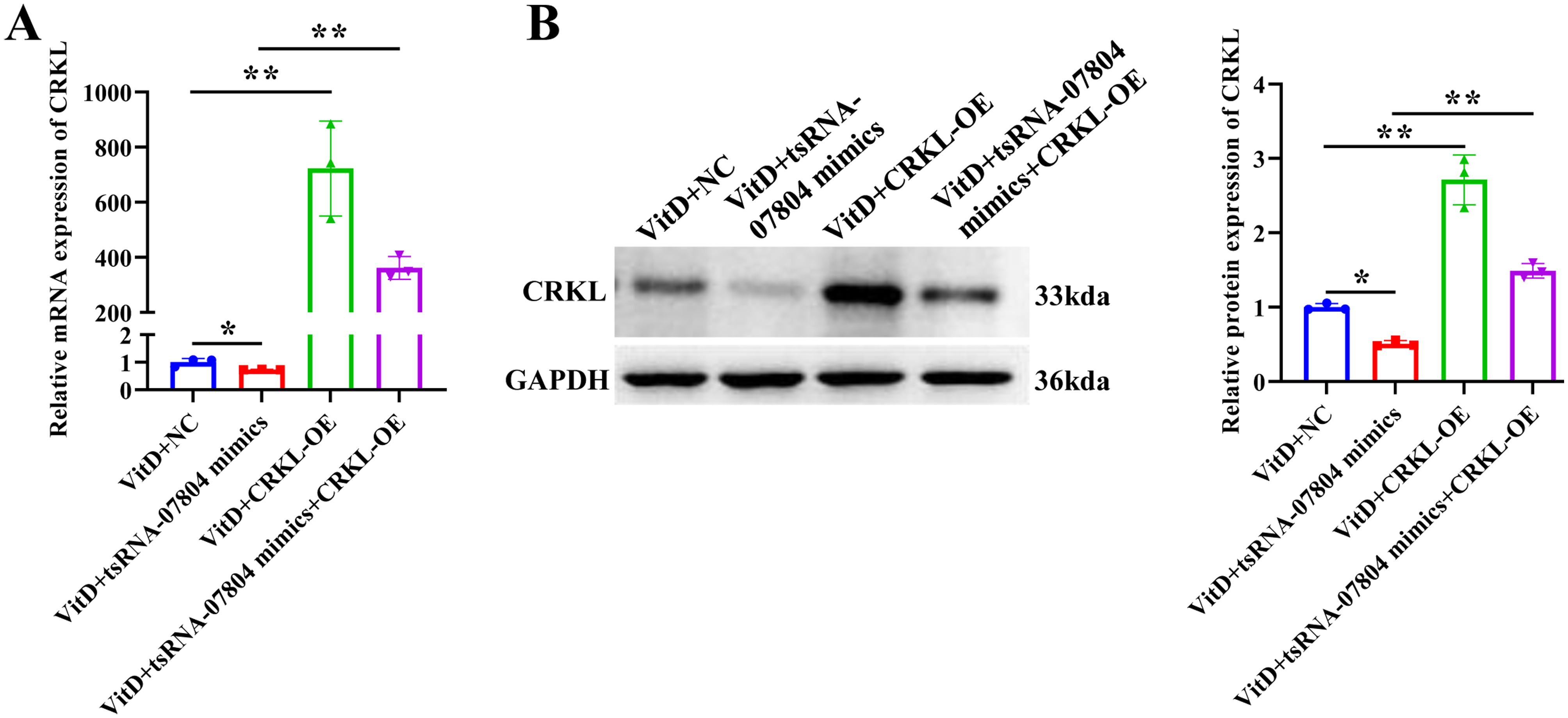

Supplement: Supplementary file 4 — Supplementary file4 Fig. S4. Overexpression of CRKL recovers the expression of CRKL in H1299 cells transfected with tsRNA-07804 mimics. The effect of tsRNA-07804 overexpression and CRKL overexpression on CRKL expression were assayed by qRT-PCR (A) and western blot (B). *P<0.05, **P<0.01. (TIF 855 KB) [file 432_2023_5586_MOESM4_ESM.tif]

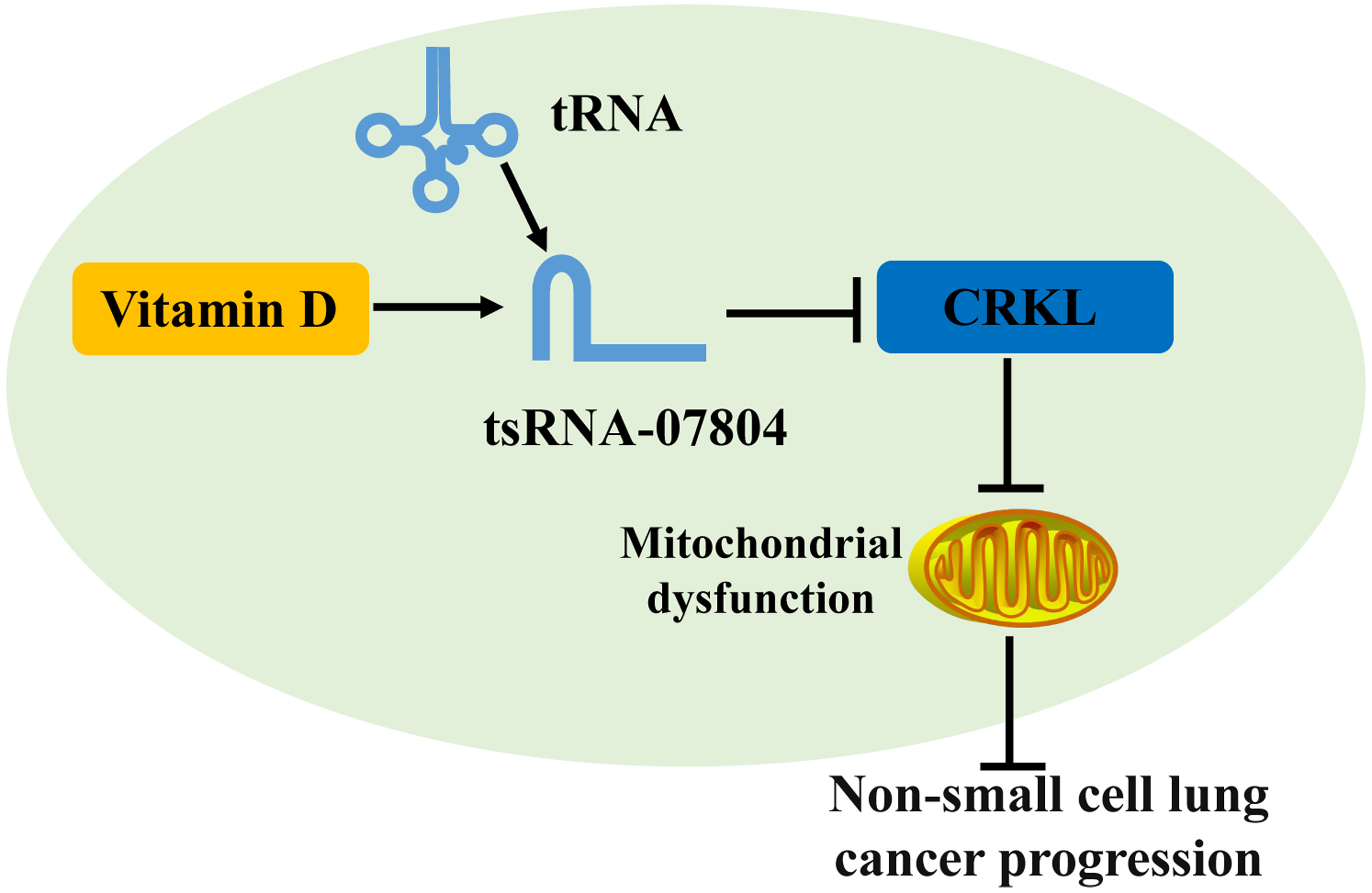

Supplement: Supplementary file 5 — Supplementary file5 Fig. S5. Schematic depiction of the rle and regulatory mechanism of vitamin D-mediated tsRNA-07804 in NSCLC. (TIF 1261 KB) [file 432_2023_5586_MOESM5_ESM.tif]
