# Supplementary material for: Vitamin D-mediated tsRNA-07804 triggers mitochondrial dysfunction and suppresses non-small cell lung cancer progression by targeting CRKL
Source: J Cancer Res Clin Oncol. 2024 Jan 30;150(2):51. doi: 10.1007/s00432-023-05586-1 (PMC10827823; doi:10.1007/s00432-023-05586-1)
Supplement: Supplementary file 6 — Supplementary file6 (DOCX 14 KB) [file 432_2023_5586_MOESM6_ESM.docx]

**Table S1. The clinicopathological features of patients with NSCLC.**

| Features | Number |
| --- | --- |
| Gender |  |
| Male | 11 |
| Female | 5 |
| Age (year) |  |
| <65 | 5 |
| ≥65 | 11 |
| TNM |  |
| Ⅰ-Ⅱ | 12 |
| Ⅲ-IV | 4 |
| Lymphatic metastasis |  |
| Absent | 12 |
| Present | 4 |
| Vascular invasion |  |
| Absent | 13 |
| Present | 3 |
| Tumor size (cm) |  |
| <5 | 13 |
| ≥5 | 3 |
| Tumor location |  |
| Left lung | 7 |
| Right lung | 9 |
