# Supplementary material for: Vitamin D-mediated tsRNA-07804 triggers mitochondrial dysfunction and suppresses non-small cell lung cancer progression by targeting CRKL
Source: J Cancer Res Clin Oncol. 2024 Jan 30;150(2):51. doi: 10.1007/s00432-023-05586-1 (PMC10827823; doi:10.1007/s00432-023-05586-1)
Supplement: Supplementary file 7 — Supplementary file7 (DOCX 14 KB) [file 432_2023_5586_MOESM7_ESM.docx]

| Primers | Sequence (5' to 3') |
| --- | --- |
| U6-F | CGATACAGAGAAGATTAGCATGGC |
| U6-R | AACGCTTCACGAATTTGCGT |
| GAPDH-F | AGAAGGCTGGGGCTCATT |
| GAPDH-R | AGAAGGCTGGGGCTCATT |
| tsrna-25432-F | ctgaaaatgtttagacgggct |
| tsrna-25432-RT | GTCGTATCCAGTGCGTGTCGTGGAGTCGGCAATTGCACTGGATACGACATGTGAG |
| tsrna-26249-F | gaaaatgtttagacgggctca |
| tsrna-26249-RT | GTCGTATCCAGTGCGTGTCGTGGAGTCGGCAATTGCACTGGATACGACGGGTGAT |
| tsrna-07804-F | gtttagacgggctcacatc |
| tsrna-07804-RT | GTCGTATCCAGTGCGTGTCGTGGAGTCGGCAATTGCACTGGATACGACTTTATGG |
| tsrna-09143-F | aatgtttagacgggctcac |
| tsrna-09143-RT | GTCGTATCCAGTGCGTGTCGTGGAGTCGGCAATTGCACTGGATACGACTGGTGTT |
| tsrna-07417-F | tgtttagacgggctcaca |
| tsrna-07417-RT | GTCGTATCCAGTGCGTGTCGTGGAGTCGGCAATTGCACTGGATACGACGGTGTTT |
| JUN-F | TTGCACTGAGTGTGGCTGAA |
| JUN-R | GACTATACTGCCGACCTGGC |
| SMAD2-F | TTCAGTTCCGCCTCCAATCG |
| SMAD2-R | GCAAGCCACGCTAGGAAAAC |
| STAT5B-F | GAACACCCGCAATGATTACAGT |
| STAT5B-R | ACGGTCTGACCTCTTAATTCGT |
| CRKL-F | CAATCACCCCTTTGCCATCC |
| CRKL-R | GACGTGCGTAAAGGGGAAAA |
| AXIN2-F | AGCCAAAGCGATCTACAAAAGG |
| AXIN2-R | AAGTCAAAAACATCTGGTAGGCA |
| LV3-NC | TTCTCCGAACGTGTCACGT |
| LV3-tsRNA-07804 inhibitor sponge | TGTTTAGACGGGCTCACATCACCCCAT |

**Table S2. Primer sequences used in qRT-PCR.**
